# Appendiceal adenocarcinoma diagnosed by endoscopic retrograde appendicitis therapy – the first clinical experience

**DOI:** 10.1055/a-2727-0064

**Published:** 2025-11-05

**Authors:** Ning Su, Jiyu Zhang, Qingfen Zheng, Lixia Zhao, Bingrong Liu

**Affiliations:** 1 191599 Department of Gastroenterology and Hepatology, The First Affiliated Hospital of Zhengzhou University, Zhengzhou, China

A 67-year-old male was admitted to our hospital with intermittent abdominal pain. Physical examination revealed no tenderness at McBurneyʼs point but tenderness around the umbilicus. CT imaging showed a dilated appendix, intraluminal fluid, and wall thickening ( Fig. 1 **a** ).

**Fig. 1:**
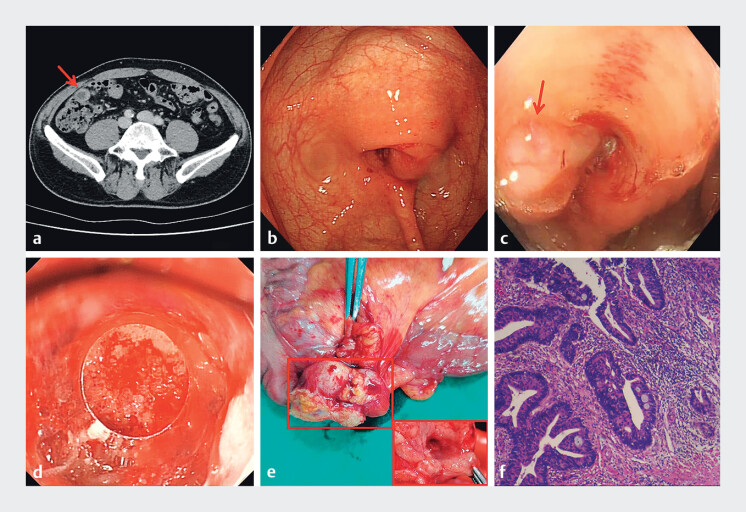
**a** CT imaging showed a dilated appendix, intraluminal fluid, and wall thickening. **b** The appendiceal orifice showed an eccentric bulge, severe edema, and mucoid secretion. **c** There is a jelly-like secretion in the appendix cavity. **d** Adenomatous hyperplasia tissue was observed at the opening of the appendiceal. **e** Radical right hemicolectomy was performed. **f** Pathological image of the surgical resection specimen.

Based on these findings and the patient’s requirement, endoscopic retrograde appendicitis therapy (ERAT) was performed. The colonoscope was advanced to the terminal ileum, revealing an eccentric bulge, severe edema, and mucoid secretion at the appendiceal orifice ( Fig. 1 **b, c** ). A guidewire was inserted using a cone-shaped transparent cap, followed by catheter insertion. A large cavity was seen via appendiceal radiography, and no fecalith was found. Given the large opening of the appendix, a conical transparent cap was used directly for irrigation and enlargement. The secretions were mixed with masses resembling necrotic tumor tissue. After repeated irrigating with normal saline, adenomatous hyperplasia tissue was observed at the opening of the appendiceal ( Fig. 1 **d** ). A biopsy was taken, which confirmed the presence of adenocarcinoma. The patient subsequently underwent radical right hemicolectomy ( Fig. 1 **e** ). Pathological examination showed vascular invasion present, neural invasion absent, no regional lymph node metastasis, and negative surgical margins ( Fig. 1 **f** ). The patient's symptoms resolved postoperatively, and 1-year follow-up imaging showed no recurrence ( Video 1 ).

The whole diagnosis and treatment process of the patient.Video 1

To our knowledge, this may represent the first reported case of appendiceal adenocarcinoma diagnosed via ERAT. Beyond its established therapeutic role, ERAT demonstrates significant diagnostic potential by enabling direct endoscopic visualization of the appendiceal orifice – a capability that positions it as a potential gold standard for detecting appendiceal orifice lesions. Our experience, based on this case, suggests that ERAT might transform the diagnostic paradigm for appendiceal pathology, potentially improving patient outcomes through earlier detection and more precise planning.

Endoscopy_UCTN_Code_TTT_1AQ_2AJ

## Notes

Endoscopy E-Videos https://eref.thieme.de/e-videos
*E-Videos* is an open access online section of the journal *Endoscopy* , reporting on interesting cases and new techniques in gastroenterological endoscopy. All papers include a high-quality video and are published with a Creative Commons CC-BY license. Endoscopy E-Videos qualify for HINARI discounts and waivers and eligibility is automatically checked during the submission process. We grant 100% waivers to articles whose corresponding authors are based in Group A countries and 50% waivers to those who are based in Group B countries as classified by Research4Life (see: https://www.research4life.org/access/eligibility/ ). This section has its own submission website at https://mc.manuscriptcentral.com/e-videos .

